# Editorial: Biopsychosocial Protective Factors Related to Antisocial Behaviors

**DOI:** 10.3389/fpsyg.2021.757355

**Published:** 2021-09-17

**Authors:** Martha Frías Armenta, Ana M. Martín, Nadia Saraí Corral-Frías

**Affiliations:** ^1^Law Department, University of Sonora, Hermosillo, Mexico; ^2^Departamento de Psicología Cognitiva, Social y Organizacional, Universidad de La Laguna, San Cristóbal de La Laguna, Spain

**Keywords:** antisocial behaviors, biological, social, psychological, protective factors

Antisocial behaviors have been associated with both direct and indirect health and social costs such as incarceration, physical injury to property and people, as well as psychological issues that affect victims, perpetrators, and their families. A myriad of biological, social, and psychological factors have demonstrated predictive utility for antisocial behaviors suggesting the multidimensional nature that should consider not only risk, but also, protective factors. Indeed, strictly investigating risk fails to consider the way protective factors may prevent antisocial behavior. Initiatives aimed at reducing antisocial behaviors thus require a multifaceted research approach that considers both risk and protective factors that can provide a framework for prevention as well as promotion of prosocial behaviors. Studies of environmental and social factors associated with family, peer groups, and neighbors have demonstrated predictive utility for psychological factors. Similarly, biological indicators from genetics, neurobiology, and endocrinology have demonstrated fruitful results. However, less focus has been paid to biopsychosocial protective factors and how these may manifest and shield against troublesome outcomes perhaps due to the relative complexity of defining and measuring these factors. This complexity requires, not only a multidisciplinary approach, but also for novel methodologies in the study of the interaction between biological, social, and psychological factors. Therefore, this Research Topic encouraged papers investigating the protective factors associated with antisocial behaviors. It included work ranging from biological, social, and psychological factors as well as the interaction between them. In “Moderating Effect of Family Support on the Mediated Relation Between Negative Life Events and Antisocial Behavior Tendencies *via* Self-Esteem Among Chinese Adolescents,” Gao et al. explored the mediating effect of self-esteem between negative life events and antisocial behavior tendencies as well as the moderated mediating effect of family support in 8,958 Chinese adolescents. The results highlight the relevance of self-esteem and family support in reducing the effects of negative life events and the prevention of antisocial behavior. Bender and Lösel in their “Adrenocortical Activity and Aggressive Behavior in Children: A Longitudinal Study on Risk and Protective Effects” paper examined, through longitudinal data collected in Germany, the effect of anxiety, family stressors, and adrenocortical activity as risk and/or protective factors in the development of antisocial behavior. The authors demonstrate the complex relationship between biological, social, and psychological factors showing, for instance, that cortisol was associated with anxiety (both cross-sectionally and longitudinally), but not with aggression. However, although stress was associated with aggression, cortisol, and anxiety, interactions between variables were not significant. Based on these findings, the authors highlight the necessity of utilizing diverse methods in the study of aggression. In a sample of Spanish participants García-García et al., in their manuscript “Emotional Assessment in Spanish Youths with Antisocial Behavior,” examined if the perception of type of image (pleasant, neutral, or unpleasant) differed by group (adolescents in juvenile justice centers, adolescents under non-custodial measures, and secondary school students) using the International Affective Picture System (IAPS). The study suggests unpleasant pictures with violent and/or aggressive content tended to differentiate antisocial from the non-antisocial groups. This study highlights the importance of experimentally examining emotions to understand the development of antisocial behaviors in adolescents. Hernandez et al. in their paper “Discounting, Cognitive Inflexibility, and Antisocial Traits as Predictors of Adolescent Drug Involvement” examined the relationship between antisocial traits, cognitive inflexibility, and loss discounting in adolescents who have had or have not used drugs. The study, which included a Mexican sample, demonstrated evidence that high antisocial traits and cognitive inflexibility differentiated those who reported drug use from those that did not. Risk-taking did not discriminate effectively between moderate consumption and problematic consumption of drugs. This study further highlights the importance of experimental tasks, this time using delay discounting, in the study of antisocial and other risk behaviors in adolescents. Finally, Frías-Armenta and Corral-Frías in their paper “Positive University Environment and Agreeableness as Protective Factors Against Antisocial Behavior in Mexican University Students” sought to investigate the effect of positive school environments and agreeableness as protective factors against antisocial behaviors in a sample of Mexican undergraduate and graduate students. Results suggest that a positive school environment helps to protect against the appearance of antisocial behavior via mood and anxiety disorders. This study highlights the importance of examining person/environment interactions by providing evidence that those with low agreeableness benefited from positive environments, whereas those with high agreeableness did not report antisocial behaviors despite being in a negative environment. In sum, this special topic includes articles from four countries (China, Spain, Germany, and Mexico) across three continents with a focus on biological (cortisol), environmental (family, stress, and university environment), and personal factors (agreeableness, self-esteem, emotions, anxiety, and cognitive flexibility) that may protect against the appearance of antisocial behavior. Results stemming from this Research Topic suggests an interplay between biological, personal, and environmental factors. Future research should replicate and extend these studies with various diverse samples. Further, policies should attend to these factors and the interplay between them to protect children and youth from antisocial behavior.

## Author Contributions

All authors listed have made a substantial, direct and intellectual contribution to the work, and approved it for publication.

## Conflict of Interest

The authors declare that the research was conducted in the absence of any commercial or financial relationships that could be construed as a potential conflict of interest.

## Publisher's Note

All claims expressed in this article are solely those of the authors and do not necessarily represent those of their affiliated organizations, or those of the publisher, the editors and the reviewers. Any product that may be evaluated in this article, or claim that may be made by its manufacturer, is not guaranteed or endorsed by the publisher.

